# Spectral Analysis and Sensitive Waveband Determination Based on Nitrogen Detection of Different Soil Types Using Near Infrared Sensors

**DOI:** 10.3390/s18020523

**Published:** 2018-02-09

**Authors:** Shupei Xiao, Yong He, Tao Dong, Pengcheng Nie

**Affiliations:** 1 College of Biosystems Engineering and Food Science, Zhejiang University, Hangzhou 310058, China; 180312@zju.edu.cn (S.X.); dt2016@zju.edu.cn (T.D.); npc2012@zju.edu.cn (P.N.); 2 Key Laboratory of Spectroscopy Sensing, Ministry of Agriculture, Zhejiang University, Hangzhou 310058, China; 3 State Key Laboratory of Modern Optical Instrumentation, Zhejiang University, Hangzhou 310058, China

**Keywords:** soil nitrogen, NIR sensors, sensitive wavebands, PLS, BIPLS, BPNN, CARS

## Abstract

Compared with the chemical analytical technique, the soil nitrogen acquisition method based on near infrared (NIR) sensors shows significant advantages, being rapid, nondestructive, and convenient. Providing an accurate grasp of different soil types, sensitive wavebands could enhance the nitrogen estimation efficiency to a large extent. In this paper, loess, calcium soil, black soil, and red soil were used as experimental samples. The prediction models between soil nitrogen and NIR spectral reflectance were established based on three chemometric methods, that is, partial least squares (PLS), backward interval partial least squares (BIPLS), and back propagation neural network (BPNN). In addition, the sensitive wavebands of four kinds of soils were selected by competitive adaptive reweighted sampling (CARS) and BIPLS. The predictive ability was assessed by the coefficient of determination *R^2^* and the root mean square error (RMSE). As a result, loess ($0.93<R_{p}^{2}<0.95,0.066\;g/\mathrm{kg}<{\mathrm{RMSE}}_{p}<0.075\;g/\mathrm{kg}$) and calcium soil ($0.95<R_{p}^{2}<0.96,\;0.080\;g/\mathrm{kg}<{\mathrm{RMSE}}_{p}<0.102\;g/\mathrm{kg}$) achieved a high prediction accuracy regardless of which algorithm was used, while black soil ($0.79<R_{p}^{2}<0.86,\;0.232\;g/\mathrm{kg}<{\mathrm{RMSE}}_{p}<0.325\;g/\mathrm{kg}$) obtained a relatively lower prediction accuracy caused by the interference of high humus content and strong absorption. The prediction accuracy of red soil ($0.86<R_{p}^{2}<0.87,\;0.231\;g/\mathrm{kg}<{\mathrm{RMSE}}_{p}<0.236\;g/\mathrm{kg}$) was similar to black soil, partly due to the high content of iron–aluminum oxide. Compared with PLS and BPNN, BIPLS performed well in removing noise and enhancing the prediction effect. In addition, the determined sensitive wavebands were 1152 nm–1162 nm and 1296 nm–1309 nm (loess), 1036 nm–1055 nm and 1129 nm–1156 nm (calcium soil), 1055 nm, 1281 nm, 1414 nm–1428 nm and 1472 nm–1493 nm (black soil), 1250 nm, 1480 nm and 1680 nm (red soil). It is of great value to investigate the differences among the NIR spectral characteristics of different soil types and determine sensitive wavebands for the more efficient and portable NIR sensors in practical application.

## 1. Introduction

Soil is the main resource supporting crop nutrients, and its spectral reflection characteristics are some of the most important features relating to its physical and chemical properties. The nitrogen content of soil directly influences the crop growth status, which is an important reference factor for crop growth estimation [1,2]. NIR spectroscopy is a common analytical method with the advantages of fast analysis speed, simple sample pretreatment, easy operation, and low cost. Compared with the traditional analytical technique, the application of NIR sensors to estimate soil nitrogen content accurately and rapidly shows a greater advantage and a wider application prospect [3,4,5].

Domestic and foreign scholars have explored the effects of soil water content, organic matter, nitrogen, phosphorus, potassium, texture, surface roughness, and pH value on soil nitrogen detection. Demattê et al. [6] used a laboratory spectroradiometer (450–2500 nm) to determine soil classes (collected from São Paulo State) by evaluating the spectral reflectance curves at different depths, and found that organic matter, total iron, silt, sand, and mineralogy were the primary factors influencing spectral intensity and characteristics. Shao et al. [7] applied least-squares support vector machines (LS-SVM) to construct calibration models for soil properties such as available nitrogen (N), phosphorus (P), and potassium (K), whose correlation coefficients of the prediction set were 0.90, 0.88, and 0.89, respectively. He et al. [8] used the NIR sensors to estimate the N, P, K, organic matter (OM) and pH content in a loamy mixed soil which were then analyzed by principal component analysis and partial least square. The results showed that the correlation coefficient between measured and predicted values of N, OM, and pH were 0.93, 0.93, and 0.91, respectively, but P and K could not be predicted.

Moreover, some researchers have demonstrated the strong correlation between soil properties and spectral reflectance characteristics using different analytical methods. Aitkenhead et al. [9] predicted the chemical and physical properties of soil by an established neural network model compared to the national soil database of Scotland. The inputs of the prediction model included soil color, texture classification, and site information while the outputs were soil organic matter, magnesium, calcium and nickel content, total alkali saturation, and pH value. The results showed that organic matter, Mg, Ca, Ni, total base saturation, and pH were predicted with a high degree of accuracy. Gao et al. [10] adopted successive projection algorithms to analyze 85 soil samples after contribution values and six characteristic wavelength values were obtained. The correlation coefficient of the prediction set was 0.913 and the prediction root mean square error (RMSEP) was 0.011%. Rinnan et al. [11] tested the applicability of NIR spectroscopy and fluorescence spectroscopic techniques in analyzing the chemical and microbial characteristics of soil during a long-term climate change manipulation experiment. The results suggested that the chemical composition and spectral properties of soil changed with the variation of the climate situation and NIR spectroscopy could be used to detect soil organic matter and fungal biomass.

In addition, the accurate determination of sensitive wavebands based on different soil types helps to improve the detection efficiency to a large extent. Zhang et al. [12] determined six sensitive wavebands (1375, 1520, 1861, 2100, 2286, and 2387 nm) for predicting the soil’s total nitrogen content using combined wavelet analysis and continuum removal technology, which achieved great accuracy in predicting the soil’s total nitrogen content in real time. Pan et al. [13] selected the sensitive wavebands of the NIR spectral range from 1692 nm to 2138 nm using moving window partial least square (MWPLS) and Savitzky–Golay (S–G) smoothing methods. The correlation coefficient of the modeling set was 0.931 and the correlation coefficient of the validating set was 0.882. Dalal et al. [14] determined the best prediction waveband of soil moisture, organic carbon, and total nitrogen using NIR diffuse reflectance spectrophotometry (1100–2500 nm) combined with multiple regression analysis. It was found that the soil’s total nitrogen characteristic wavebands were 1702, 1870, and 2052 nm. Lu et al. [15] analyzed the spectral reflectance variation of black soil in Northeast China between 350 and 2500 nm by the reflectance logarithm of the first derivative spectrum, and determined that the optimum prediction model of black soil total nitrogen content and the selected bands were 556, 1642, and 2491 nm, whose *R*^2^ was 0.863 and RMSE was 0.122 g/kg.

The objective of this study was to compare the influences of soil types and modeling methods on soil nitrogen detection by NIR sensors as well as determine the optimum sensitive wavebands of four kinds of soils. In addition, it was to investigate the potential of replacing full wavebands by sensitive wavebands in soil nitrogen prediction, which is helpful for improving the overall detection efficiency and developing more efficient and professional instruments for soil nitrogen detection.

## 2. Materials and Methods 

### 2.1. Experimental Materials

The experimental materials included loess, calcium soil, black soil, and red soil collected from various regions, whose nitrogen content differs from each other, which is of great credibility to investigate the difference of NIR spectra taking those four kinds of soils for experimental samples. The loess was collected from Xi’an (34°27′ N, 108°95′ E, Shannxi province, China), and was mainly comprised of detrital mineral (including quartz, feldspar, and carbonate mineral) and clay mineral. The proportion of coarse particles (0.05–0.01 mm) in loess is more than 50% with high carbonate content, and the content of organic matter and nitrogen in loess is relatively low due to geographical distribution. The calcium soil was collected from Jining (35°23′ N, 116°33′ E, Shandong province, China), and contained a certain amount of organic matter and ash with high carbonate content (calcium carbonate and magnesium carbonate). The soil texture was loose and the humus layer was thick. The black soil was collected from the Greater Khingan Mountains (46°08′ N, 122°05′ E, Inner Mongolia Autonomous Region, China), and was neutral or slightly alkaline with organic content between 3% and 10%. The black soil mainly included calcareous sedimentary rock, basic igneous rock, basalt, and volcanic ash, resulting in the characteristics of having high organic matter as well as being one of the most fertile soils in China. The red soil was collected from Lishui (27°62′ N, 119°05′ E, Zhejiang province, China), having the characteristics of high water content, low density, and high intensity. It was mainly comprised of carbonate and rock rich in iron–aluminum oxides weathered in the hot and humid climate.

The sample preparation process was as follows: First, the four kinds of soils were all collected from the surface layer 0–20 cm from the ground, and were sieved with a 40-mesh sieve (0.425 mm). Second, the urea solutions with different nitrogen concentrations were prepared and mixed with soil samples where the nitrogen concentration ranges were loess (0.09–0.93 g/kg, 176 samples), calcium soil (0.33–1.17 g/kg, 176 samples), black soil (0.47–2.15 g/kg, 176 samples) and red soil (0.47–2.10 g/kg, 144 samples). Meanwhile, the four kinds of soils without urea added were set as references. Third, all soil samples were compressed into slices by an acrylic plate (PMMA) and divided into easily detectable sizes, then dried in an oven at 80 degrees Celsius for 24 h in order to eliminate the interference of soil moisture on NIR spectra.

### 2.2. NIR Spectrum Collection

The characteristic signal of the NIR spectral region can reflect the main structure and composition of various organic compounds with the advantages of being stable and easy to handle [16,17]. The NIR spectrum collection experiment used a portable NIR optical spectrum instrument (NIRez) from Isuzu Optics Corp (Shanghai, China), which is reflective with two integrated tungsten halogen lamps. This instrument collects spectral information in the range of 900–1700 nm; its optical resolution is 10 nm, and the signal–noise ratio is 5000:1 in a one-second scan. Before performing the spectral determination, the instrument should be preheated for 15 min and be prepared with blackboard and whiteboard correction operations. During the spectrum acquisition process, the spectral scan range, scale rate, and scan times can be set as required, and the information about intensity, reflectance, and absorbance of the spectrum can be collected. This experiment collected 400 points of each spectrum and obtained a spectral image at an average of 3 times per scan. In this experiment, 672 soil samples of four kinds of soils were measured in total. 

### 2.3. Preprocessing of Spectral Data

Savitzky–Golay (S–G) smoothing was used to preprocess the raw spectral data in this experiment, quantizing the spectral data in the moving window by polynomial least squares fitting as well as emphasizing the central role of the center point. The formula of average wavelengths after S–G smoothing is [18,19]:

(1) $$x_{k,smooth}=\bar{x_{i}}=\frac{1}{H}\overset{+w}{\underset{i=-w}{\sum }}x_{k+1}hi$$

where *H* is the normalization factor, hi is the smoothing coefficient, and $H=\sum_{I=-W}^{+W}h_{i}$. The measured value multiplied by the smoothing coefficient is to minimize the smoothing influence on the useful information. In this experiment, the original NIR spectra were processed with 9-point S–G smoothing.

### 2.4. Chemometrics Methods

In this experiment, three chemometric methods (PLS, BIPLS, BPNN) were used to establish the prediction model between soil nitrogen content and NIR spectra. Standard Normal Variation (SNV) was also included to correct the baseline in PLS, BIPLS, and BPNN, whose principle is that the absorbance values of each wavelength point satisfy a certain distribution in each spectra, and the spectral correction is performed according to this assumption [20]. In addition, the sensitive wavebands of four types of soils were determined by CARS and BIPLS. In this paper, all data analysis was based on MATAB R2014a (The Math-Works, Natick, MA, USA).

#### 2.4.1. Partial Least Squares

Partial least squares (PLS) is a regression modeling method of multi-dependent variables to multi-independent variables, which extracts the most comprehensive variables and identifies the noise by decomposing and filtering the data in the system. When there is a high correlation within each set of variables, PLS can compare multiple variables by multiple regression to establish a more reliable model [21,22]. When PLS is applied to the spectral data model, the spectral matrix is decomposed first, the main principal component variables are obtained, and the contribution rate of each principal component is calculated. Based on the accumulative contribution rate of principal components, the first few principal components are selected as input to establish a mapping relationship with chemical indicators. Generally, the flexibility of PLS makes it possible to establish a regression model in the case where the number of samples is less than the number of variables, which is one of the least restrictive methods in the multivariate correction method [23]. 

#### 2.4.2. Backward Interval Partial Least Squares

Backward interval partial least squares (BIPLS) is a variable selection method mainly used to filter the wavelength range of the PLS model and reduce the amount of sub-intervals of the worst or collinear variables. It selects the best principal component number according to the root mean square error of cross validation (RMSECV) [24,25]. The algorithm steps are mainly as follows:

1. Divide the whole spectrum into k bands of equal width;
2. Reserve a section from the k section spectrum, carry out PLS regression on the remaining (k-1) section, and establish the sub-model of the quality to be measured. Set aside each paragraph in order to get the k sub-model;
3. Evaluate the precision of each model through the RMSECV value, delete the reserved segment according to the sub-model with the highest precision, and take the sub-model as the first base model;
4. Leave one more section in the remaining (k-1) section of the spectrum and use the remaining (k-2) segments to model the PLS. Each section is set aside in order to obtain the (k-1) sub-model to remove the reserved segments corresponding to the sub-model of the minimum RMSECV value; take the sub-model as the second base model. Repeat the above process until there is a remaining waveband.
5. Examine the RMSEP value of each base model according to steps 2 to 4, select the best and minimum RMSECV among all the base models, and the corresponding interval combination is the best combination.

#### 2.4.3. Back Propagation Neural Network

The back propagation neural network (BPNN) is a supervised learning algorithm in an artificial neural network (ANN) [26]. It is a multilayer feedforward network which corrects the weights by the error propagation algorithm. The ANN model simulates the neural structure of the brain based on the processing network of large numbers of processing units to train and model the data. The basic idea of the BPNN algorithm is to iteratively optimize the weight of the network and establish the mapping relationship between the dependent variables and the independent variables [27]. It calculates the error of each layer by calculating the error of each unit and adjusts the weights by the gradient descent algorithm, in order to improve the training speed and obtain the optimum objective function. In addition, the BPNN algorithm can approximate the arbitrary function in theory. Its basic structure is composed of non-linear variable elements; it has a strong nonlinear mapping ability, learning adaptability, and fault tolerance, and has a wide application prospect in data mining and artificial intelligence.

#### 2.4.4. Competitive Adaptive Reweighted Sampling

Competitive adaptive reweighted sampling (CARS) is a feature wavelength selection method based on Monte Carlo sampling and the PLS regression coefficient, which eliminates the unadapted wavelength variables by the random sampling method [28]. This algorithm chooses part of the samples in the total sample set to carry out PLS modeling, and in the process of iterating over hundreds of times, only the wavelength variables with a large absolute value for the PLS regression coefficient remained and the wavelength variables with a small absolute value for the PLS regression coefficient were removed, aiming to obtain a part of the optimal wavelength variable subset [29]. In this experiment, CARS was used to select the characteristic variables for the further determination of sensitive wavebands.

### 2.5. SPXY Method

SPXY is the abbreviation of sample set partitioning based on joint x–y distance, which was put forward by Galvao et al [30] based on (Kennard-Stone)K-S methods. The basic idea of this method is to consider the spectral characteristics and physicochemical properties at the same time by calculating the distance between the samples. The distance formula is as follows:

(2) $$d_{xy}=\frac{d_{x}\left(i,j\right)}{max_{i,j\in \left(i,j\right)}\left[d_{x}\left(i,j\right)\right]}+\frac{d_{y}\left(i,j\right)}{max_{i,j\in \left(i,j\right)}\left[d_{y}\left(i,j\right)\right]},i,j\in \left[1,z\right]$$

where $d_{x}\left(i,j\right)$ refers to the spectral characteristic parameters for the calculation of the distance between the samples while $d_{y}\left(i,j\right)$ refers to concentration characteristic parameters for the calculation of the distance between the samples. The $d_{x}\left(i,j\right)$ and $d_{y}\left(i,j\right)$ were divided by their corresponding maximum values, which makes the sample in spectrum space and concentration space have the same weightiness. z is the spectral space.

### 2.6. Model Evaluation Index

In this experiment, the modeling effect was evaluated by the coefficient of determination *R^2^* and the root mean square error (RMSE). The coefficient of determination *R^2^* reflects the level of intimacy between variables while the RMSE reflects the accuracy of the model. The closer the *R^2^* to 1 and the closer the RMSE to 0, the better the performance of the prediction model. In this paper, $R_{c}^{2}\;$ and $R_{p}^{2}$ represent the coefficient of determination of the calibration set and the prediction set, respectively. ${\mathrm{RMSE}}_{c}$ and ${\mathrm{RMSE}}_{p}$ represent the root mean square error of the calibration set and the prediction set, respectively. In addition, all data processing was based on MATLAB R2014a (The Math-Works, Natick, MA, USA).

## 3. Results and Discussion

### 3.1. Analysis of Soil NIR Spectrum

**Figure 1:**
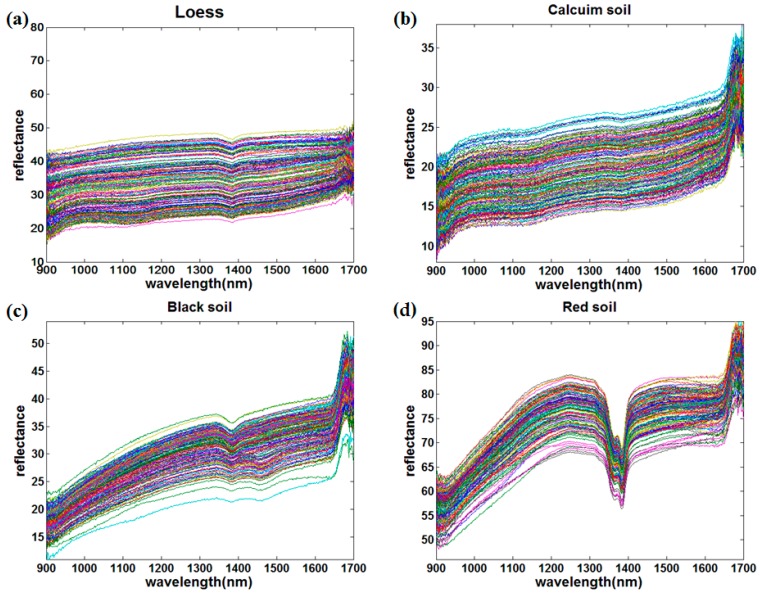
The original near infrared (NIR) curves of four kinds of soils. (**a**) Loess; (**b**) Calcium soil; (**c**) Black Soil; (**d**) Red soil.

**Figure 2:**
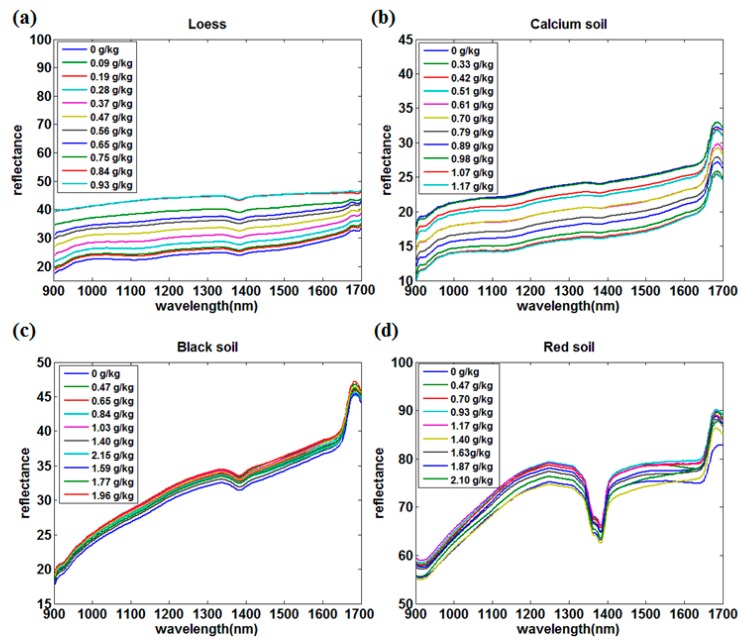
The average NIR spectral curves of four kinds of soils at each nitrogen concentration. (**a**) Loess; (**b**) Calcium soil; (**c**) Black Soil; (**d**) Red soil.

This study collected the NIR spectra of four kinds of soils by a portable NIR sensor. The original spectral curves of the four kinds of soils are shown in Figure 1 while the average spectral curves preprocessed by the S–G smoothing of the four kinds of soils at different nitrogen concentrations are shown in Figure 2. The abscissa is the wavelength and the ordinate is the spectral reflectance, and each curve represents the variation in the average spectral reflectance of the soil sample with the wavelength at a certain nitrogen concentration.

Comparing the original and average spectra of the four kinds of soils, there are many differences in the peak, reflectance, and dispersion degree. First, the absorption peaks of the red soils were the most obvious in the range of 1350 nm to 1380 nm, followed by black soil and loess, while the absorption peak of the calcium soil was the weakest. Second, the dispersion degree of spectral curves from large to small were loess, calcium soil, red soil, and black soil. Third, there were also large differences in reflectance among the different types of soils using NIR spectroscopy. The reflectance of loess and calcium soil changed slightly within the whole wavelength range, where the reflectance of loess was in the range of 15 to 45 and the calcium soil was 10 to 35. In addition, the reflectance increased slowly with the increase of soil nitrogen concentrations and wavelength, where the reflectance of the same soil nitrogen concentration curve increased by only 10 within the whole wavelength range, which was similar to calcium soil. The reflectance of black soil and red soil changed significantly, where the variation range was 15 to 50 and 55 to 90, respectively. Although the reflectance range of black soil was similar to that of loess, the reflectance increasing extent of the same curve within the whole wavelength range was much larger than that of the loess, which was around 30. The reflectance of red soil was in the range of 55 to 90, which was the highest among the four soil types, and its overall trend was similar to that of black soil.

### 3.2. Full-Waveband Data Analysis and Modeling

In this experiment, three chemometric methods were used to establish the prediction models between soil nitrogen content and the NIR spectrum. The four kinds of soil samples were divided into two groups according to the ratio of the calibration set and the prediction set of 2:1 using the SPXY method.

#### 3.2.1. Partial Least Squares

**Figure 3:**
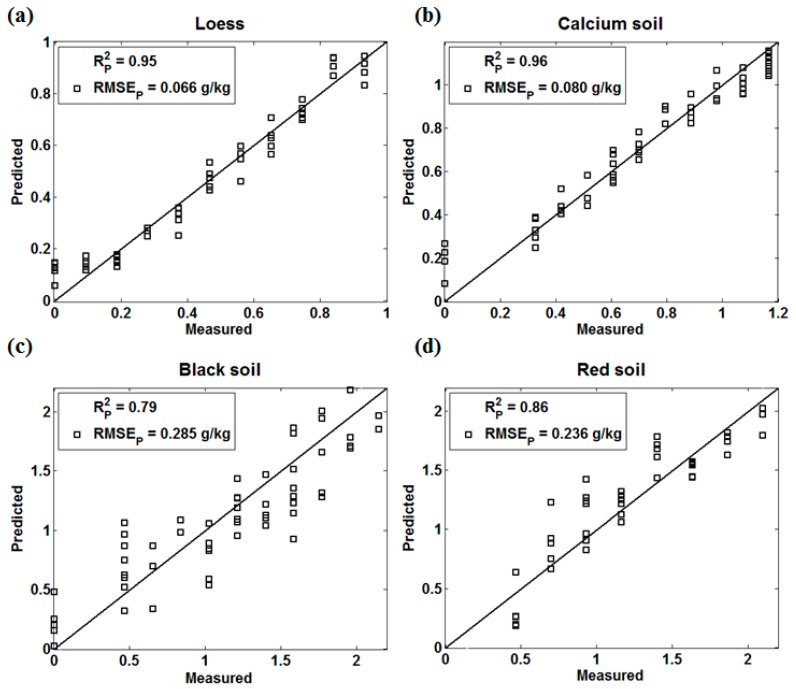
The Partial Least Squares (PLS) effect of four kinds of soils. (**a**) Loess; (**b**) Calcium soil; (**c**) Black Soil; (**d**) Red soil.

In this experiment, the NIR spectral matrix was the independent variable X, and the soil nitrogen concentration was the dependent variable Y. Figure 3 presents the prediction performance of the four kinds of soils by PLS.

PLS is the most commonly used chemometrics modeling method, which has the least restrictive conditions during usage. In the process of PLS modeling, when the noise of the NIR spectrum has a great influence on the selection of principal components, the prediction performance is largely influenced by soil properties. Zhang et al. [31] analyzed the relationship between the total nitrogen content and the NIR spectral reflectance of five soil types (paddy soil, fluvo-aquic soil, salinized fluvo-aquic soil, saline soil, and black soil with lime concretion), where black soil with lime concretion and salinized fluvo-aquic soil showed the worst prediction performance. In our results, the PLS prediction models of loess, calcium soil, and red soil achieved a good effect, especially loess ($R_{p}^{2}=0.95,{\;\mathrm{RMSE}}_{p}=\;0.066\;g/\mathrm{kg}$) and calcium soil ($R_{p}^{2}=0.96,{\;\mathrm{RMSE}}_{p}=\;0.080\;g/\mathrm{kg}$). The prediction accuracy of black soil ($R_{p}^{2}=0.79,{\;\mathrm{RMSE}}_{p}=\;0.236\;g/\mathrm{kg}$) was lower than that of the other three soils, which was consistent with Zhang’s research results. The reason might be that the absorption of the organic matter in black soil is strong, which has a certain influence on both the NIR spectrum and the selection of the principal component.

#### 3.2.2. Backward Interval Partial Least Squares

**Figure 4:**
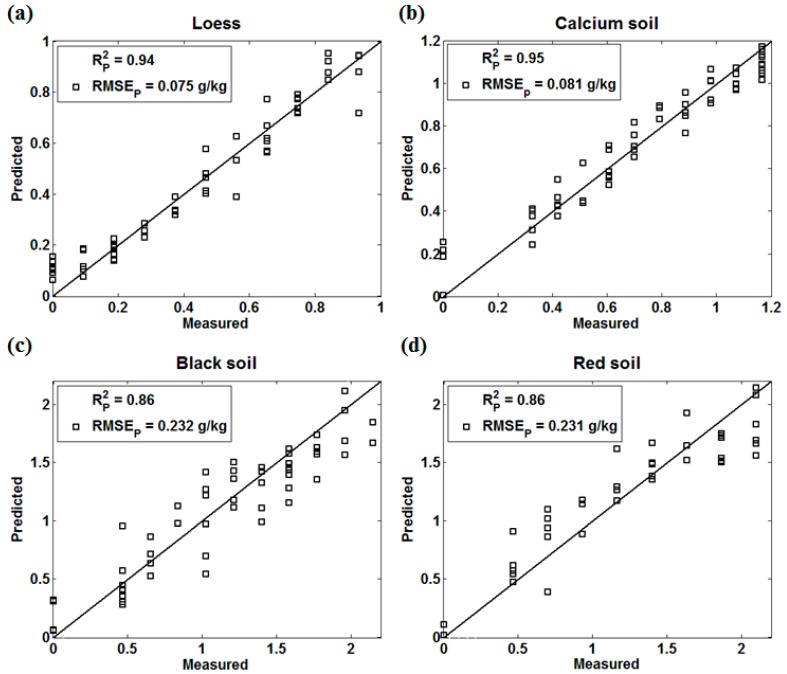
The Backward Interval Partial Least Squares (BIPLS) effect of four kinds of soils (**a**) Loess; (**b**) Calcium soil; (**c**) Black Soil; (**d**) Red soil.

Backward interval partial least squares is based on the interval least squares, which removes the maximum interval of RMSECV and establishes the optimal PLS model based on the remaining interval. When the RMSECV is the smallest and the corresponding interval is the optimized combination, the model with poor information can optimize the modeling effect to a certain extent for the collinearity variable. The prediction effect of four kinds of soils by BIPLS was shown in Figure 4. 

Loess ($R_{p}^{2}=0.94,{\;\mathrm{RMSE}}_{p}=\;0.075\;g/\mathrm{kg}$), calcium soil ($R_{p}^{2}=0.95,{\;\mathrm{RMSE}}_{p}=\;0.081\;g/\mathrm{kg}$), and red soil ($R_{p}^{2}=0.86,{\;\mathrm{RMSE}}_{p}=\;0.231\;g/\mathrm{kg}$) displayed a similar prediction performance as well as those in PLS models. However, the black soil ($R_{p}^{2}=0.86,{\;\mathrm{RMSE}}_{p}=\;0.232\;g/\mathrm{kg}$) with poor prediction accuracy in PLS achieved a better result using the BIPLS algorithm, which suggested that the BIPLS algorithm might be able to eliminate the interference noise caused by the strong absorption of black soil and reduce the collinear variables to enhance the prediction accuracy.

#### 3.2.3. Back Propagation Neural Network

**Figure 5:**
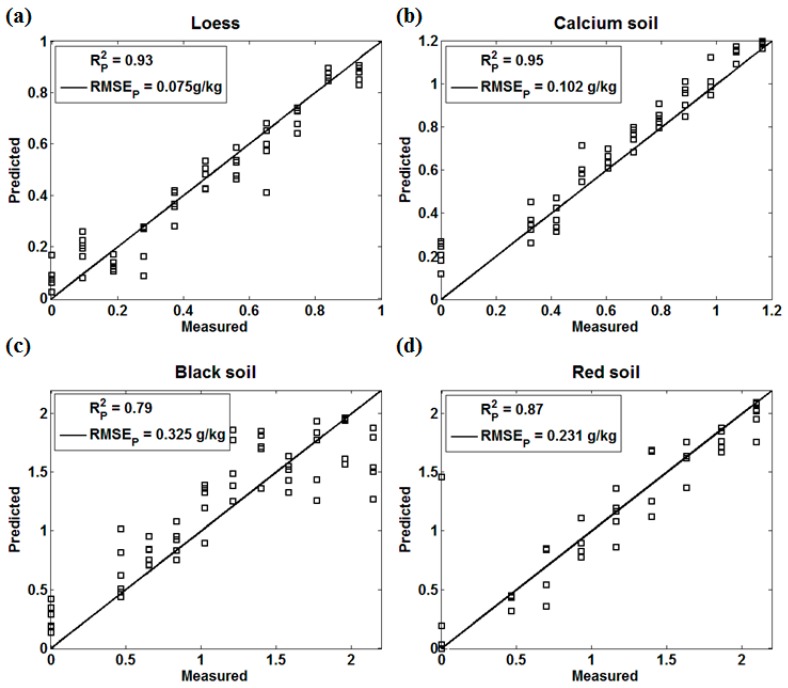
The Back Propagation Neural Network (BPNN) effect of four kinds of soils (**a**) Loess; (**b**) Calcium soil; (**c**) Black Soil; (**d**) Red soil.

Back Propagation Neural Network is the most representative and widely used neural network, with the advantages of a simple structure and strong operability, and it can simulate arbitrary nonlinear input and output relations. Figure 5 presents the prediction effect of four kinds of soils by the BPNN model. 

In this algorithm, the *mapminmax* function was used to normalize and de-normalize the BP neural network model of 3-layer network topology. The *tansig* function was used as the hidden layer transfer function, *purelin* was the output layer function and *trainlm* was the training function [32]. The learning rate was set as 0.1, the maximum number of training times was 1000, and the model expectation error was 0.00005. While running this algorithm, the iteration number was set at 100 times, considering both the modeling effect and algorithm running efficiency. 

Loess ($R_{p}^{2}=0.93,{\;\mathrm{RMSE}}_{p}=\;0.075\;g/\mathrm{kg}$), calcium soil ($R_{p}^{2}=0.95,{\;\mathrm{RMSE}}_{p}=\;0.102\;g/\mathrm{kg}$), black soil ($R_{p}^{2}=0.79,{\;\mathrm{RMSE}}_{p}=\;0.325\;g/\mathrm{kg}$), and red soil ($R_{p}^{2}=0.87,{\;\mathrm{RMSE}}_{p}=\;0.231\;g/\mathrm{kg}$) all achieved similar prediction effects in BPNN models as well as those in PLS models. The BPNN algorithm has a strong nonlinear mapping ability to approach the best objective function, but fails to eliminate the interference information before training and modelling [27]. The results indicated that both PLS and BPNN had a relatively poor performance in predicting the nitrogen content of black soil affected by the nature of black soil itself.

### 3.3. Sensitive Wavebands Selection

#### 3.3.1. The Selection Process of Sensitive Wavebands

In this paper, the CARS algorithm was used to select the characteristic variables of the NIR spectrum whose sampling times were set to 500, while the BIPLS algorithm was used to select the characteristic intervals. The red soil was an example to express the selection process of characteristic variables and characteristic intervals.

**Figure 6:**
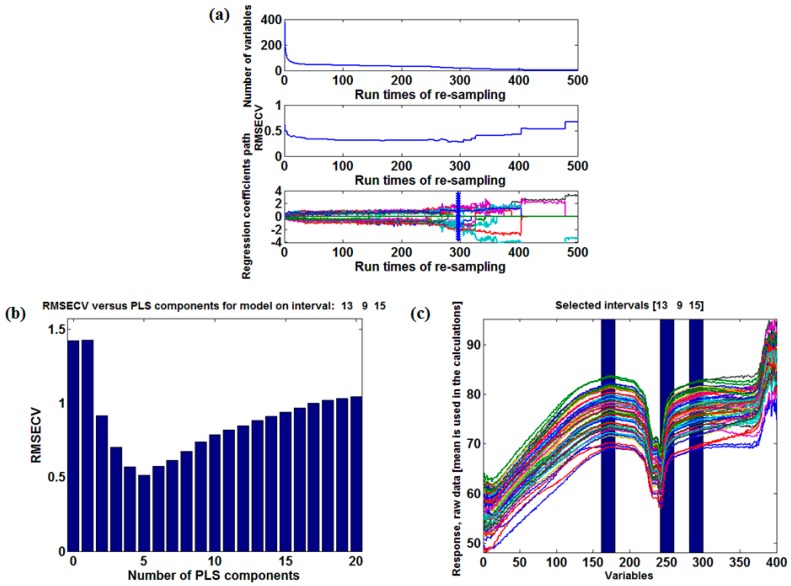
The sensitive waveband selection process (**a**) The characteristic variables selected by Competitive Adaptive Reweighted Sampling (CARS); (**b**) The number of PLS components and the corresponding Root Mean Square Error of Cross Validation (RMSECV) values while running BIPLS; (**c**) The characteristic intervals determined by BIPLS.

Figure 6a displays the selection process of characteristic variables carried by CARS. While running the CARS algorithm, the variables were selected by attenuation functions during each iteration and only the wavelength variables with large absolute values of PLS regression coefficients were preserved. According to the running results, the characteristic variables that occurred with high frequency were selected and grouped while running the CARS algorithm.

Figure 6b shows the characteristic intervals of the soil spectrum selected by BIPLS and Figure 6c presents the best principal numbers determined by leave-one-out cross validation. Table 1 lists the variable selection results for BIPLS when the spectral intervals are divided into 20 segments. As the spectral intervals are gradually removed by BIPLS, the RMSECV values change constantly and the number of remaining intervals and variables in the model decrease. When the spectral interval of number 11 is removed, the RMSECV of the model reaches a minimum value of 0.233, and the number of removed intervals is 14. The remaining three intervals in the model are 13, 9, and 15, which have participated in the final modeling, and the number of variables in the model is 60.

Table 2 presents the characteristic variables selected by CARS and the characteristic intervals selected by BIPLS of four kinds of soils. Linear correlation analysis was performed for each characteristic variable and each characteristic interval, and the results were sorted by correlation coefficients from the highest to the lowest. Finally, the characteristic variables and the characteristic intervals with high correlation were determined as the sensitive wavebands of soil according to the principle of the least number of variables and the best prediction effect. As a result, the determined sensitive wavebands were 1152 nm–1162 nm and 1296 nm–1309nm (loess), 1036 nm–1055 nm and 1129 nm–1156 nm (calcium soil), 1055 nm, 1281 nm, 1414 nm–1428 nm and 1472 nm–1493 nm (black soil), 1250 nm, 1480 nm and 1680 nm (red soil).

According to the Beer–Lambert law, the NIR spectrum varies with the variation of sample components and structure, and different groups generate different positions and intensities of spectral peaks [33]. Zhang et al. [12] selected six sensitive wavebands (1375, 1520, 1861, 2100, 2286, and 2387 nm) for detecting soil nitrogen. Yang et al. [34] obtained the least collinear effective wavelength (820, 940, 1040, 1060, and 1990 nm) of red soil using the successive projections algorithm (SPA) for variable selection. Kawamura et al. [2] suggested that the reflectance values at 1413 and 2207 nm were highly correlated with the soil’s total carbon and total nitrogen contents. Although the determination of sensitive wavebands was influenced by soil type, detection instrument, and the spectral preprocessing method, it is partly similar in some waveband range. Therefore, it is of great application value to replace full wavebands with sensitive wavebands for improving the detection efficiency of soil nitrogen.

#### 3.3.2. Modeling Effect of Sensitive Wavebands

**Figure 7:**
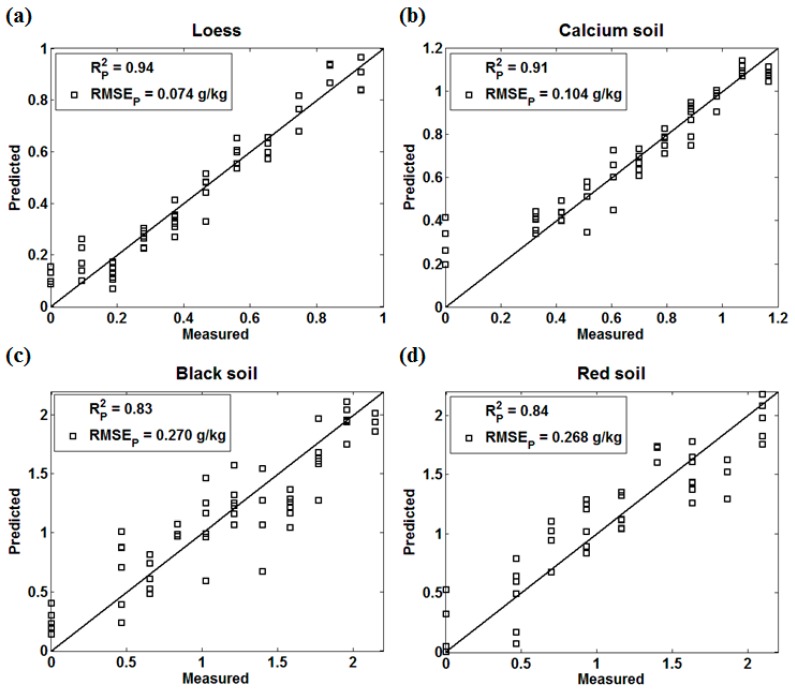
The sensitive waveband modeling effect of four kinds of soils (**a**) Loess; (**b**) Calcium soil; (**c**) Black Soil; (**d**) Red soil.

The sensitive wavebands of four kinds of soils were determined combining the results of characteristic variables and characteristic intervals. The prediction effect of sensitive wavebands modeled by PLS is shown in Figure 7.

The PLS prediction model established by sensitive wavebands of four kinds of soils achieves relatively good prediction accuracy as well as full wavebands, especially for loess ($R_{p}^{2}=0.94,{\;\mathrm{RMSE}}_{p}=\;0.070\;g/\mathrm{kg}$) and calcium soil ($R_{p}^{2}=0.91,{\;\mathrm{RMSE}}_{p}=\;0.104\;g/\mathrm{kg}$). The black soil ($R_{p}^{2}=0.83,{\;\mathrm{RMSE}}_{p}=\;0.270\;g/\mathrm{kg}$) modeled by sensitive waveband performed better than that of PLS and BPNN but slightly worse than that of BIPLS. Both BIPLS and sensitive waveband partly eliminate the interference noise in the NIR spectrum of black soil, which enhances the prediction accuracy to a large extent. The prediction accuracy of red soil ($R_{p}^{2}=0.84,{\;\mathrm{RMSE}}_{p}=\;0.268\;g/\mathrm{kg}$) was slightly worse than that of PLS, BIPLS, and BPNN. Although the sensitive wavebands removed most of the interference noise, soil properties would affect the soil nitrogen detection using NIR sensors as well. However, the sensitive wavebands alternative is highly feasible to improve the modeling efficiency instead of the full-waveband spectrum, and it is a good prospect applicable to different soil types.

### 3.4. Comparison of Modeling Effect

In this experiment, PLS, BIPLS, and BPNN were used to establish the prediction model between the NIR spectrum and soil nitrogen content. CARS and BIPLS were used to determine the sensitive wavebands of four kinds of soils, and the prediction models of sensitive wavebands were established by PLS. In addition, the modeling effect of different soil types, different models, and sensitive wavebands were compared. The coefficient of determination *R*^2^ and RMSE of the calibration set and prediction set are shown in Table 3.

From the perspective of soil properties, regardless of which prediction model (PLS, BIPLS, and BPNN) was used, both loess and calcium soil achieved good prediction accuracy. The reason is that the N–H bonds in the soil mostly exist in multiple frequency or combination frequency, while the vibration of the O–H and C–H bonds is stronger than that of the N–H bonds, which is one of the main factors affecting the NIR spectrum of soil nitrogen. The main components of loess are ${\mathrm{SiO}}_{2}$, ${\mathrm{Al}}_{2}O_{3}$, and CaO while the main component of calcium soil is ${\;\mathrm{CaCO}}_{3}$ [35]. Both loess and calcium soil have few O–H bonds after drying which interferes with the NIR spectrum to a large extent. Hence, the prediction accuracies of loess and calcium soil were the best among the four kinds of soils. The black soil and red soil obtained relatively low prediction accuracy compared with loess and calcium soil. Soil color reflects spectral characteristics in the visible light band, which is closely related to physical and chemical properties such as soil organic matter content, iron oxide content, texture, clay content, and moisture, which dominated clay mineral types [36]. The high humus content in black soil [37] and the strong absorption resulted in the worst prediction accuracy among the four kinds of soils. Although the color of red soil is not as dark as black soil, the high $\mathrm{Fe}{\left(\mathrm{OH}\right)}_{3}$ and $\mathrm{Al}{\left(\mathrm{OH}\right)}_{3}$ content in red soil [38] indicates more O–H bonds. The strong vibration of O–H bonds affected the vibration of the N–H bonds in the soil, which resulted in a poor prediction effect as well.

From the perspective of algorithms, both loess and calcium soil achieved the highest $R_{p}^{2}$ (0.95 and 0.96) and the lowest ${\mathrm{RMSE}}_{p}$ (0.066 g/kg and 0.080 g/kg) in PLS models, indicating that PLS models could achieve a relatively good modeling effect for soils with less interference noise. The BIPLS model of black soil ($R_{p}^{2}=0.91,{\;\mathrm{RMSE}}_{p}=\;0.104\;g/\mathrm{kg}$) obtained the best prediction performance among the three algorithms, and it performed well in removing the interference noise caused by the strong absorption of black soil itself. The three models of red soil achieved similar prediction effects and reached the best performance in the BIPLS model as well as black soil, proving that BIPLS has a good effect on removing the interference noise and enhancing the prediction accuracy.

## 4. Conclusions

In this paper, the NIR spectral characteristics of loess, calcium soil, black soil, and red soil were investigated and three chemometric methods (PLS, BIPLS, and BPNN) were used to establish the prediction model between the NIR spectrum and soil nitrogen content. In addition, the sensitive wavebands of four kinds of soils were determined by BIPLS and CARS.

The principal results obtained can be summarized as follows: (1) The sensitive wavebands of four kinds of soils were determined, that is, 1152 nm–1162 nm and 1296 nm–1309nm (loess), 1036 nm–1055 nm and 1129 nm –1156 nm (calcium soil), 1055 nm, 1281 nm, 1414 nm–1428 nm and 1472 nm–1493 nm (black soil), 1250 nm, 1480 nm and 1680 nm (red soil); (2) The prediction performance evaluated by *R^2^* and RMSE from good to bad was calcium soil ($0.95<R_{p}^{2}<0.96,\;0.080\;g/\mathrm{kg}<{\mathrm{RMSE}}_{p}<0.102\;g/\mathrm{kg}$), loess ($0.93<R_{p}^{2}<0.95,\;0.066\;g/\mathrm{kg}<{\mathrm{RMSE}}_{p}<0.075\;g/\mathrm{kg}$), red soil ($0.86<R_{p}^{2}<0.87,\;0.231\;g/\mathrm{kg}<{\mathrm{RMSE}}_{p}<0.236\;g/\mathrm{kg}$) and black soil ($0.79<R_{p}^{2}<0.86,\;0.232\;g/\mathrm{kg}<{\mathrm{RMSE}}_{p}<0.325\;g/\mathrm{kg}$), and the O–H bonds in soil might be the main factor influencing the prediction accuracy; (3) The PLS and BPNN models obtained a better prediction effect for soil with less noise, while BIPLS performed well in removing the interference noise caused by the strong vibration of O–H or absorption of soil. 

## Figures and Tables

**Table 1:** The processing results of characteristic variables by BIPLS.

Interval Number	Selected Interval	RMSECV	Variable Numbers	Interval Number	Selected Interval	RMSECV	Variable Numbers
20	6	0.274	400	10	3	0.255	200
19	16	0.270	380	9	1	0.255	180
18	19	0.266	360	8	20	0.254	160
17	7	0.265	340	7	12	0.246	140
16	17	0.262	320	6	10	0.241	120
15	8	0.256	300	5	11	0.236	100
14	5	0.255	280	4	14	0.233	80
13	4	0.254	260	3	13	0.240	60
12	2	0.253	240	2	9	0.260	40
11	18	0.254	220	1	15	0.435	20

Intervals number, the number of intervals in the model; Selected interval, the interval selected by the BIPLS; RMSECV, root mean square error cross validation; Variable numbers, the number of variables in the model.

**Table 2:** The selected characteristic variables, characteristic intervals, and sensitive wavebands.

Soil Type	CARS Algorithm	BIPLS Algorithm		Sensitive Wavebands (nm)
	Characteristic Variables (nm)	Characteristic Intervals (nm)	Serial Number of Characteristic Interval	
Loess	1152–1162	1036–1078, 1250–1329, 1411–1448, 1487–1523, 1561–1596	4, 8, 9, 10, 13, 15, 17	1152–1162, 1296–1309
Calcium Soil	1129–1159	900–944, 1080–1290, 1525–1559	1, 4, 5, 6, 7, 8, 9, 16, 20	1036–1055, 1129–1156
Black Soil	1414–1429, 1472–1493	1036–1078, 1250–1590, 1411–1559	4, 9, 12, 13, 14, 15, 16	1055, 1281, 1414–1428, 1472–1493
Red soil	1464–1469, 1480, 1680	1225–1290, 1441–1447, 1486–1523	9, 13, 15	1250, 1480, 1680

**Table 3:** The *R*^2^ and RMSE values of four kinds of soils (loess, calcium soil, black soil and red soil) modeled by full wavebands (PLS, BIPLS, BPNN) and sensitive wavebands (PLS).

Soil Type	Model	Calibration Set		Prediction Set		
		$R_{c}^{2}$	$RMSE_{c}$(g/kg)	$R_{p}^{2}$	$RMSE_{p}$(g/kg)	The Tested Range (g/kg)
Loess	PLS	0.95	0.066	**0.95**	0.066	0.057–0.944
	BIPLS	0.93	0.079	0.94	0.075	0.063–0.950
	BPNN	0.96	0.053	0.93	0.075	0.025–0.909
	CARS/BIPLS-PLS	0.92	0.080	0.94	0.074	0.041–0.972
Calcium Soil	PLS	0.98	0.045	**0.96**	0.080	0.043–1.247
	BIPLS	0.95	0.074	0.95	0.081	0.018–1.173
	BPNN	0.96	0.038	0.95	0.102	0.101–1.196
	CARS/BIPLS-PLS	0.88	0.158	0.91	0.104	0.242–1.255
Black Soil	PLS	0.98	0.086	0.79	0.285	0.079–2.149
	BIPLS	0.88	0.212	**0.86**	0.232	0.069–2.111
	BPNN	0.94	0.167	0.79	0.325	0.133–1.970
	CARS/BIPLS-PLS	0.85	0.249	0.83	0.270	0.240–2.112
Red Soil	PLS	0.90	0.210	0.86	0.236	0.196–2.025
	BIPLS	0.87	0.239	0.86	0.231	0.028–2.133
	BPNN	0.95	0.158	**0.87**	0.231	0.019–2.090
	CARS/BIPLS-PLS	0.86	0.249	0.84	0.268	0.014–2.166
